# *Curcuma Longa* (turmeric): from traditional applications to modern plant medicine research hotspots

**DOI:** 10.1186/s13020-025-01115-z

**Published:** 2025-05-28

**Authors:** Wei-Wei Tian, Li Liu, Ping Chen, Dong-Mei Yu, Qing-Miao Li, Hua Hua, Jun-Ning Zhao

**Affiliations:** 1 Key Lab.: Biological Evaluation of TCM Quality of the State Administration of Traditional Chinese Medicine, Sichuan Engineering Technology Research Center of Genuine Regional Drug, Sichuan Provincial Engineering Research Center of Formation Principle and Quality Evaluation of Genuine Medicinal Materials, Translational Chinese Medicine Key Laboratory of Sichuan Province, Sichuan Institute for Translational Chinese Medicine, Chengdu, 610041 China; 2 Sichuan Academy of Chinese Medical Sciences, Chengdu, 610041 China; 3 National Center for Nanoscience and Technology, Beijing, 100190 China

**Keywords:** *Curcuma longa* L*.*, Traditional applications, Pharmacological activities, Clinical applications, Products

## Abstract

Turmeric, derived from the dried rhizome of *Curcuma longa* L*.*, receives widespread attention because of its applications in pharmaceutical, food, cosmetic and other industries. Traditionally, it has been widely used in Ayurveda medicine and traditional Asian medicine such as traditional Chinese medicine, for treatment of digestive, respiratory and circulatory diseases, as well as skin diseases. However, a comprehensive review of traditional applications, modern clinical applications, and related products remains largely unexplored. Here, we conduct a systematic summary of its pharmacological activities, including anti-inflammatory activity, anti-oxidant activity, anti-diabetic activity, anti-tumor activity, neuroprotective activity, hepatoprotective activity, anti-microbial activity and others. Additionally, we explore the randomized controlled trials, guiding future preventive healthcare strategies and clinical practices. Furthermore, we also discuss the turmeric-related products, involving medicines, health foods, herbal dietary supplements, and cosmetics, offering novel insights into relevant product development. Totally, this review provides a comprehensive understanding of turmeric on botany, history and traditional applications, pharmacological activities, clinical applications, and related products. Finally, based on the generalized science of Chinese material madica and advanced front technologies, the future research opportunities of turmeric are briefly explored.

## Introduction

The genus *Curcuma*, composed of roughly 130 species, is widely distributed in tropical and subtropical areas, including China, India, Thailand, Malaysia, Indonesia, etc. [1]. Some *Curcuma* species possess medicinal, edible, and ornamental values. *Curcuma longa*, the most well-known species of the *Curcuma* genus, is grown in warm climates and cultivated in tropical and subtropical regions worldwide. It is known by multiple names across cultures such as turmeric in English, Haldi in Hindi, manjal in Tamil, kunyit in Indonesian, Jianghuang in Chinese, and Kyoo in Japanese. The medicinal history of turmeric dates back 4 000 years [2]. Turmeric has historically been used as a traditional herbal medicine in China, India, Thailand, Malaysia, Indonesia, Japan, South Korea, and other countries. Traditionally, turmeric has been utilized for treatment of respiratory, digestive, and circulatory diseases, as well as skin diseases. 

Nowadays, extensive research has confirmed that turmeric contains a variety of active ingredients, such as diphenylalkanoids, terpenoids, aromatics, steroids, fatty acids, minerals, and nucleosides. These components contribute to the treatment of inflammatory diseases, digestive diseases, cardiovascular diseases, skin diseases, cancers, etc. As traditional herbal medicine, medicinal and food homologous variety, and cosmetic ingredient, turmeric is widely used in pharmaceutical, food, cosmetic and other industries, making products such as drugs, health foods, food additives, dietary supplements, cosmetics. Representing the largest application segment, the pharmaceutical industry accounts for over 50% of the worldwide market [3]. As the main active ingredient of turmeric, curcumin market size (pharmaceutical, food and cosmetics) reached $98.7 million in 2023 and is estimated with a 9.1% compound annual growth rate by 2032. Recent advancements in curcumin extraction techniques, including high-intensity and ultrasonic-assisted water filtration, have improved both the yield and purity of curcumin, resulting in higher potency and efficacy. Novel delivery systems such as liposomes, micelles and nanoparticles significantly increase curcumin absorption and boost its therapeutic efficacy. The expanding use in diverse industries, including pharmaceutical industry, food industry, and cosmetic industry, is also contributing to the product uptake (https://www.gminsights.com/). Therefore, this review aims to encompass various applications of turmeric, including its history and traditional applications, clinical applications, and related products. Finally, based on the generalized science of Chinese material madica, the cultivation system of large variety of turmeric and the application system of the large health industry are constructed.

## Botany

**Fig. 1:**
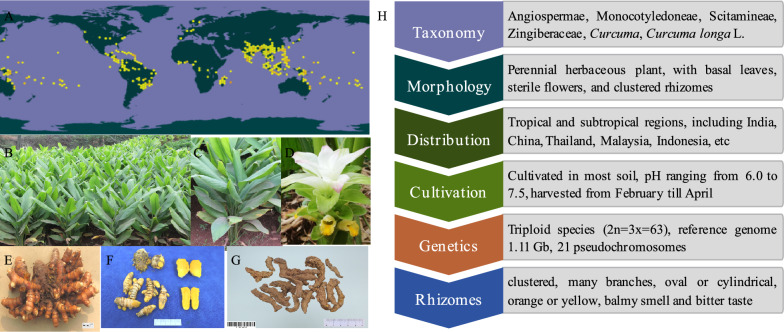
Botanical characteristics of turmeric. **A** global distribution of turmeric (www.gbif.org), **B**, **C** turmeric, **D** the flowers of turmeric, **E**, **F**, **G** the rhizomes of turmeric, **H** a brief summary of botanical characteristics of turmeric

*Curcuma longa*, a triploid specie (2n = 3x = 63), belongs to the genus *Curcuma*. Morphologically, turmeric is a perennial herb that reaches a height of approximately 1–1.5 m. The leaves are basal, usually oblong to elliptic in morphology, 30–50 cm in length and 15–18 cm in width, with dark green on the upper surface and pale green beneath. The sterile flowers present pale yellow petals with a purplish covering, complemented by green bracts with a purplish colour. The rhizomes (underground stem) are well-developed, clustered, with many branches, oval or cylindrical, orange or yellow, balmy smell and bitter taste (Fig. 1). There are many varieties, including Suguna, Sudarsana (tolerant to rhizome rot), Suroma, IISR Alleppey Supreme (resistant to leaf blotch), IISR Prabha, IISR Prathiba (high yielding variety), Co.1, BSR.1 (resistant to drought), BSR.2, Rashmi (bold rhizomes), Chuanjianghuang 1 (high productivity and adaptability variety), etc. [4]. Pheap et al. reported five *Curcuma longa* varieties collected from Siem Reap province, Cambodia, including Black ginger (BG), Broteal lakai (BLK), Broteal roneang (BRN), Sena 100 (SN1) and Fire ginger (FG). The results showed that curcumin was the main component in BG, BRN and SN1, while not detected in FG and BLK [5]. Alam et al. evaluated the rhizome yield and related traits of 53 *Curcuma longa* genotypes from 2019 to 2021. According to the yield and overall ranking, the top ten genotypes were identified as superior, such as T0129, T0121, T0117, T0106, T0103, T0094, T0085, T0082, T0061, and T0015 [6]. Yin et al. successfully constructed a high-quality genome assembly of *Curcuma longa* spanning 1.11 Gb, which provided insight into germplasm resources identification, new varieties breeding, and disease-resistant gene mining [7].

Data from the GBIF database (https://www.gbif.org/) shows that the resources of turmeric on a global scale are mainly distributed in India, China, Thailand, Singapore, Philippines, Malaysia, Indonesia, Australia, and other countries (Fig. 1). India contributes 80% of the global turmeric production [8], while China accounts for 8%, Myanmar 4%, and Nigeria and Bangladesh 3% each. Gururani et al. assessed the differences in chemical profiles and biological activities of essential oil derived from native *Curcuma longa* rhizome cultivars in Garhwal and Kumaun regions of Uttarakhand, India. The findings revealed that the quantity and composition of essential oil derived from turmeric rhizomes harvested in Garhwal and Kumaun regions of Uttarakhand exhibited variations [9]. In China, turmeric is widely cultivated in Sichuan, Yunnna, Fujian, Guangdong, Taiwan, and other proviences. Qianwei county in Sichuan provience is the main producing areas of turmeric, accounting for about 60% of national output and called “Chuan Jiang Huang”. We constructed the bioinformatics database and production layout visual analysis platform of medicinal plants, covering geographic data, phenotype data, compound data, and genetic data of turmeric in Sichuan province [10].

## History and traditional applications

**Fig. 2:**
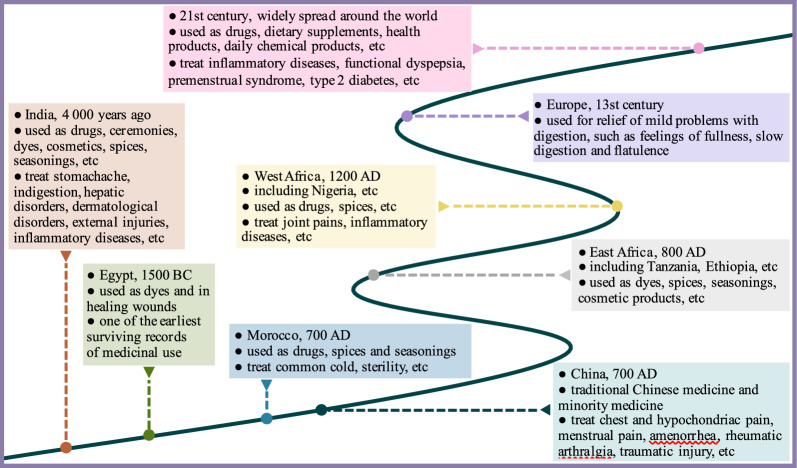
The history and traditional applications of turmeric

Turmeric has been utilized by humans for nearly 6,000 years [11]. Historically, turmeric was widely used in Ayurveda medicine and traditional Asian medicine such as traditional Chinese medicine. The exact origin of turmeric are unknown. According to records, the use of turmeric in India dated back roughly 6,000 years. It probably spread to both Morocco and China by around 700 AD, reached East Africa by 800 AD and West Africa by 1200 AD. Then in the thirteenth century, Arab merchants brought turmeric to Europe [12]. Alternatively in sixteenth century, turmeric entered Turkish cuisine, where it served as a natural coloring agent to give yellow color to the saffron-infused rice dessert [13]. Until eighteenth century, turmeric was introduced to Jamaica. Nowadays, turmeric has been widely spread around the world, and used as drugs, health foods, food additives, dietary supplements, cosmetics (Fig. 2).

### Traditional applications in India

Owing to its bright yellow color, turmeric is referred to “Indian saffron” “manjal” “haldi”. Traditionally in India, turmeric was used as drugs, religious ceremonies, dyes, cosmetics, spices, and seasonings. The medicinal use of turmeric was first documented in ‘Atharveda’. In the Ayurveda system, turmeric has been applied to cure common cold, stomachache, flatulence, indigestion, hepatic disorders, jaundice, bilious attack, gallstones, rheumatism, irregular menstruation, dermatological disorders (skin infections, pimples and foul ulcers), external injuries (sprains, wounds, swellings and cuts), inflammatory diseases (rhinitis, arthritis and inflammatory bowel disease) [14, 15]. Moreover, turmeric was used in many important ceremonies, such as weddings. Additionally, turmeric was employed to religious observances, including Hinduism and Buddhism. In Hinduism and Buddhism, turmeric powder symbolized fertility, prosperity, and purity. Further, the conventional saffron-colored robes donned by Buddhist monks was dyed with turmeric. Likewise, turmeric was traditionally used as a facial mask to enhance the complexion and reduce skin blemishes. Also known as the “Spice of life”, turmeric has been utilized for spice and food seasoning, adding flavor and color to dishes.

### Traditional applications in China

After introduced into China in the Tang Dynasty, turmeric immediately attracted widespread attention. The medicinal use of turmeric was first mentioned in ‘*New Revised of Materia Medica*’ (659 AD)*.* Subsequently, turmeric has been recorded in numerous classical ancient Chinese medicine books, such as ‘*Ri Hua-zi’s Materia Medica*’, ‘*Bencao Tujing*’, ‘*Compendium of Materia Medica*’, providing detailed information on the medicinal effects of turmeric. Traditional Chinese medicine (TCM) is mainly divided into two categories, traditional Chinese medicinal material, and traditional Chinese medicine preparation [16]. Turmeric is used as traditional Chinese medicinal material, and traditional Chinese medicine preparation. Based on the ‘*Catalogue of Ancient Classical Formulas (First Batch)*’ and ‘*Catalogue of Ancient Classical Formulas (Two Batch)*’, turmeric is widely used in TCM and minority medicine, including Tibetan medicine, Mongolian medicine, and Dai medicine (Table 1). In TCM practice, the rhizomatous and tuberous parts of *Curcuma longa* are classified as two distinct herbal medicines (named as “Jiang Huang” and “Huang Si Yu Jin”, respectively). The “Jiang Huang” is generally believed to be warm and suitable for treating chest and hypochondriac pain, menstrual pain, amenorrhea, postpartum abdominal pain, rheumatic arthralgia, traumatic injury, jaundice, etc. But the “Huang Si Yu Jin” is characterized by its cold properties and is associated with the liver, heart, and lung meridians. It is used for promoting the circulation of qi and blood, relieving pain, clearing heat and cooling the blood, as well as for its cholagogic (bile-promoting) and jaundice-alleviating effects.

**Table 1:** Catalogue of ancient classical formulas containing turmeric

Rank	Prescription name	Prescription source	Prescription compositions	Action of prescription
1	Huanglian Gao	Yizongjinjian	*Coptis chinensis, Angelica sinensis, Rehjnannia glutinosa, Phellodendron chinense, Curcuma longa*	Treat retention of heat-phlegm in the lung, xerostomia, edema and pain, eczema, erythema and swelling, thermal ulceration, burns and scalds, and mammary fissures
2	Wuwei Jianghuang Pill	Xiuduoyixuehuiji	*Curcuma longa, Phyllanthus emblica, Berberis amurensis, Thlaspi arvense, Tribulus terrestris*	Treat lumbocrural pain, turbid urine, rectal tenesmus, urinary frequency, urinary urgency caused by ‘Jingnisaku’ and ‘Kaichang’
3	Shiwei Qinglan San	Sibuyidian	*Dracocephalum tanguticum, Taraxacum mongolicum, Ribes emodens, Hippophae rhamnoides, Curcuma longa, Rhododendron primuliflorum, Cinnamomum cassia, Myristica fragrans, Polygonatum sibiricum, Tinospora sinensis*	Treat abdominal distension and pain, gastroesophageal reflux, eructation, abdominal distension, abdominal pain, constipation, and hematochezia caused by ‘Peigenmubu’
4	Pipaye erwei Decoction	Tongwagajide	*Eriobotrya japonica, Curcuma longa*	Treat menorrhagia, excessive vaginal discharge and generalized weakness
5	Yapengle	Danghayadaoxiangnen	*Curcuma longa, Zingiber montanum, Acorus calamus, Rheum franzenbachii, Artemisia argyi*	Treat gastralgia, epigastric distending pain, vomiting, and diarrhea
6	Yajiezhanla	Danghayalong	*Curcuma longa, Zingiber montanum, Nigella glandulifera, Zingiber officinale*	Treat stroke, deafness, cardiac and chest pain, hematuria, urolithiasis, sallow complexion and emaciation, abdominal distension and pain
7	Yalongjiuduanga	Danghayahemai	*Curcuma longa, Zingiber montanum, Acorus calamus, Foeniculum vulgare, Amomum kravanh, Camphora officinarum, Piper nigrum, Ferula sinkiangensis*	Treat abdominal distension and pain, nausea and vomiting, dysmenorrhea, and muscle and joint crampy pain
8	Yajieduan	Danghayamengdai	*Curcuma longa, Zingiber montanum, Acorus calamus, Artemisia argyi, Stephania cepharantha Hayata, Eclipta prostrata, Curcuma zedoaria*	Treat indigestion, abdominal distension and pain, belching and acid reflux, peptic ulcer, and gastric spasm
9	Yawalutazhuan	Danghayadaoxiangnen	*Nigella glandulifera, Curcuma longa, Zingiber montanum, Piper nigrum*	Used for the treatment of rheumatism-related myalgia, epigastric and abdominal pain, and dizziness and headache
10	Shengjiang San	Shanghanwenyitiaobian	*Bombyx mori, Cryptotympana pustulata, Curcuma longa, Rheum officinale*	–

### Traditional applications in other countries

Also, in South Asia, turmeric has been used for treatment of cuts, burns, and bruises [17]. In Japan, turmeric has been widely used for digestive disorders, and enjoyed as a tea, particularly in Okinawa [18]. In Korea, the turmeric was used as antidotes for hematuria and anxiety [19]. The ancient Hawaiians used turmeric for treatment of sinus infections, ear infections and gastrointestinal ulcers [2]. In Nigeria, turmeric was utilized as spices and herbs (for joint pain and inflammation) [20]. In Islamic medicine, powdered *Curcuma longa* extract was used to curing pimples and wounds [21]. Additionally, in Kurdistan and surrounding areas, *Curcuma longa* has been utilized in relieving joint inflammation, promoting weight management, enhancing culinary flavor, as well as exhibiting antiviral and anticancer applications [22]. Moreover, *Curcuma longa* has been used for treatment of the diseases related to blood and circulatory system, digestive system, musculoskeletal system, urinary system, etc.

## Applications in modern medical practice

### Phytochemical composition

Recently, numerous bioactive compounds have been identified through diverse analytical techniques, such as HPLC (high-performance liquid chromatography), GC–MS (gas chromatography mass spectrometry), LC–MS (liquid chromatography mass spectrometry), and NMR (nuclear magnetic resonance). Turmeric contains a variety of active pharmaceutical ingredient, including diphenylalkanoids, terpenoids, aromatics, steroids, and fatty acids. Additionally, turmeric also contains a variety of macro and micro elements, including K, Mg, Ca, Na, Al, Cr, Cu, Mn, Rb, Sr, and Zn [23]. Recent years, various reports revealed that microRNAs (miRNAs) could be the potential active ingredients and critical material foundation of traditional Chinese medicine, which have been proved to transfer across species, facilitating cross-kingdom regulation by incorporating themselves into specific target gene-driven regulatory pathways, thereby executing associated biological functions [24]. Our previous study suggested that turmeric extract contained abundant miRNAs, including 10 known and 115 novel miRNAs, predicting 13,575 target genes.

### The pharmacological activities

**Fig. 3:**
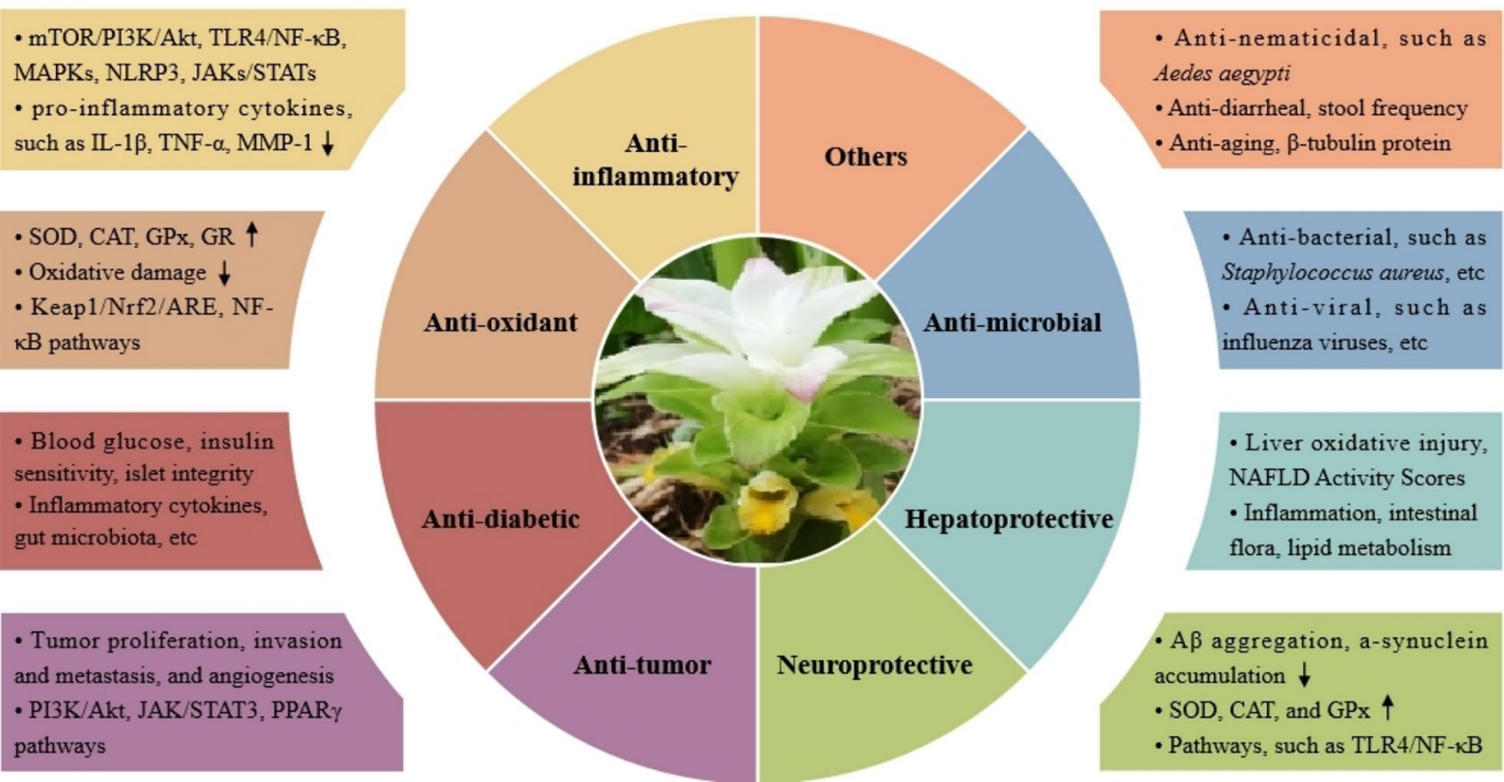
The pharmacological activities of turmeric

Modern pharmacological studies showed that turmeric have many activities, including anti-inflammatory activity, anti-oxidant activity, anti-diabetic activity, anti-tumor activity, neuroprotective activity, hepatoprotective activity, anti-microbial activity and others. The pharmacological activities were summarized in Fig. 3.

#### Anti-inflammatory activity

Generally, turmeric is recognized for exhibiting various biological activities, with anti-inflammatory activity being one of the most notable characteristics. Over the years, numerous studies confirmed that turmeric effectively inhibits multiple signaling pathways in inflammation including mTOR/PI3K/Akt, TLR4/NF-κB, MAPKs, NLRP3, and JAKs/STATs. For example, Dai et al. demonstrated that curcumin significantly ameliorated collagen-induced arthritis rat model in vivo by inhibiting the increased levels of key pro-inflammatory mediators such as TNF-α, IL-1β, MMP-1, and MMP-3 via the mTOR pathway [25]. The protective effect of curcumin against high glucose-induced inflammation in retinal pigment epithelial cells was achieved through suppression of the ROS/PI3K/AKT/mTOR pathway [26].

The transcription factor NF-κB, as a central regulator of inflammatory responses, plays a crucial role in the pathogenesis of diverse inflammatory disorders. Therefore, the NF-κB pathway provides a good choice for treatment of inflammatory diseases. In BV2 cells, curcumin suppressed LPS-induced neuroinflammation by enhancing microglial M2 polarization through mechanisms involving the TREM2/TLR4/NF-κB signaling pathways. In monosodium iodoacetate (MIA)-induced osteoarthritis rat model, curcumin possessed an anti-inflammatory effect against osteoarthritis and prevented knee damage via blocking the TLR4/NF-κB signaling pathway [27]. Furthermore, curcumin-loaded polysaccharide microparticles mitigated DSS-induced ulcerative colitis through modulation of gut microbiota and the MAPK/NF-κB/Nrf2/NLRP3 signaling axis [28].

Moreover, the essential oils extracted from the rhizome of turmeric also have anti-inflammatory activity. α-turmerone, *ar*-turmerone, and β-turmerone were the main components in essential oils, accounting for 12.9%, 42.6%, and 16.0%, respectively [29]. *ar*-Turmerone, a turmeric oil derived from turmeric, exhibited anti-inflammatory activity against Hela-STAT3-Luc cells. It possessed an inhibitory effect by activating the NF-κB and STAT3 pathways, with respective IC_50_ values of 22.7 ± 3.2 μM and 14.21 ± 4.7 μM. Further evaluation of the anti-inflammatory activity of turmeric showed that α-turmerone attenuated HIF-1α-mediated signaling by suppressing desferrioxamine-induced activation of erythropoietin promoter activity [30]. Additionally, in activated microglial cells, the inhibition of the IKK/NF-κB signaling pathway by turmeronol A and turmeronol B could potentially block the generation of inflammatory mediators [31]. 3-hydroxy-1,7-bis(4-hydroxy-phenyl)-1,3-heptadiene-5-one and bisabola-3,10-diene-2-one, also displayed anti-inflammatory activities against LPS-induced NO production in RAW264.7 cells. Both of them showed the IC_50_ values of 14.42 and 12.93 μM, respectively, compared to the positive control hydrocortisone with IC_50_ values of 37.64 μM [32]. Calebin A, a bioactive compound derived from turmeric, prevented stress-induced damage in chondrocytes by suppressing programmed cell death, extracellular matrix breakdown, and key pathways involved in inflammatory responses (NF-κB, MMP9) or inhibition of autophagy (mTOR/PI3K/Akt) [33].

#### Anti-oxidant activity

Turmeric has potential anti-oxidant activity by inhibiting reactive oxygen species (ROS) accumulation, activating antioxidant signaling pathways, and inducing oxidative damage. One study suggested that curcumin can scavenge or neutralize ROS through its phenolic OH and the β-diketone moiety [34]. A previously reported study showed that curcumin could prevent the release of mitochondrial type 1 hexokinase, a key enzyme controlling brain glucose metabolism, and induce an increase in ROS through α-synuclein fibrillation products [35]. Additionally, curcumin exerted hepatoprotective property against LPS-induced acute and chronic hepatic toxicity under stress conditions, mediated by suppressing reactive oxygen species accumulation, restoring normal endoplasmic reticulum protein folding functionality, and alleviating hepatic dyslipidemia [36]. As a key transcription factor, Nrf2 regulates the expression of numerous antioxidant genes. One study showed that curcumin enhanced the oxidative stress resistance in corneal endothelial cells by activating the Keap1/Nrf2/ARE signaling pathway [37]. Another study found that curcumin prevented cadmium or H_2_O_2_-induced oxidative stress via Nrf2/ARE signaling and autophagy in myeloid cells [38]. Another study also demonstrated that curcumin displayed renoprotection activity via activating the nuclear levels of Nrf2, reducing the nuclear activity of NF-κB, suppressing NADPH oxidase, and down-regulating PKCβII/p [66] Shc axis [39]. Besides, in SH-SY5Y cells, curcumin reduced oxidative damage caused by OGD/R through modulation of the miR-1287-5p/LONP2 pathway [40]. In the oxidative damage rat models caused by sodium arsenate, curcumin enhanced the antioxidant defense system by increasing the enzymatic activities of CAT, GR, GPx, and SOD [41].

The ethanol extract of turmeric showed scavenging activities against HO, 2,2- diphenyl-1-picrylhydrazyl (DPPH), and 2,2’-azino-bis-3-ethylbenzothiazoline-6-sulphonic acid (ABTS) [42]. The essential oil extracted from turmeric rhizomes demonstrated concentration-dependent antioxidant activity against ABTS and DPPH radicals, with respective IC_50_ values of 0.54 mg/mL and 10.03 mg/mL [43]. In vivo mouse models of myocardial infarction, by regulating Nrf2-SIRT3 pathway, tetrahydrocurcumin alleviated oxidative stress as well as mitochondrial damage [44]. Furthermore, the 1:1 mixture of dimethylmethoxy chromanol and turmeric root extract reduced ultraviolet-induced oxidative damage in HaCaT cells via cooperative enhancement of cellular antioxidant enzyme systems [45].

#### Anti-diabetic activity

Diabetes impacted 529 million individuals globally in 2021, with estimates projecting this number will surge to 1.31 billion by 2050 [46]. Turmeric exhibits potent anti-diabetic activity via suppressing oxidative stress and inflammatory process [47]. Three curcuminoids, including curcumin, demethoxycurcumin, and bisdemethoxycurcumin, significantly reduced blood glucose, alanine aminotransferase, and aspartate aminotransferase levels, and improved liver histopathology score, indicating that these three curcuminoids have potent anti-diabetic efficacy [48]. Zhong et al. ameliorated insulin resistance, glucose intolerance, triglyceride accumulation, and pyruvate intolerance in the liver of mice on a high-fat diet by modulating gut microbiota [49]. In addition, curcumin supplementation enhanced the hepatic expression of insulin-degrading enzyme and maintained the structural integrity of pancreatic islets [50]. It has been demonstrated that 15 μM of curcumin could induce preadipocyte apoptosis and inhibit adipocyte differentiation. This mechanism is associated with the down-regulation of PPARγ and CCAAT enhancer binding proteins, the prevention of differentiation medium-induced down-regulation of β-catenin, and a reduction lipid accumulation in 3T3-L1 adipocytes [51]. Two other studies proved that curcumin regulated lipid metabolism and suppressed chronic inflammation by targeting white adipose tissue, playing a key role in addressing obesity-related health issues [52, 53]. Additionally, in a high-fat diet-induced obesity mouse model, dietary intervention with curcumin demonstrated an ability to alleviate metabolic disease in vivo. This effect was mediated by the prevention of uncoupling protein 1 expression in brown adipose tissue and the modulation of macrophage functional polarity in white adipose tissue [54].

#### Anti-tumor activity

Recently, turmeric has gained considerable attention due to its notable anti-tumor activity. Many studies have demonstrated that curcumin is the main anti-tumor active ingredient derived from turmeric. It exhibits significant anti-tumor activity in treating multiple cancers, such as breast cancer, cervical cancer, colorectal cancer, lung cancer, papillary thyroid cancer, etc. Jin et al. found that curcumin suppressed cell proliferation and induced apoptosis in vitro, with the underlying mechanism involving the activation of miR-192-5p and inhibition of the PI3K/Akt pathway [55]. Additionally, in B-CPAP cells, curcumin promoted apoptosis and suppressed proliferation by inhibiting lncRNA LINC00691, potentially via modulation of the Akt signaling pathway [56]. In addition, in colorectal cancer cells, curcumin inhibited colorectal cancer metastasis by activation of the ROS/KEAP1/NRF2/miR-34a/b/c pathway. Moreover, the therapeutic efficacy of 5-FU against p53- and miR-34a/b/c-deficient colorectal cancer cells may be potentiated by curcumin [57]. Another study demonstrated that curcumin suppressed the proliferation, metastasis, epithelial mesenchymal transition, and stem cell-like properties in triple-negative breast cancer through modulation of the Hedgehog/Gli1 signaling cascade [58]. Angiogenesis is one of the important mechanisms of the occurrence, development, invasion, and metastasis of tumors. Jin et al. indicated that the combination of curcumin and (-)-epigallocatechin-3-gallate inhibited angiogenesis triggered by the colorectal cancer microenvironment through modulation of the JAK/STAT3/IL-8 signaling axis [59]. Moreover, curcumin suppressed angiogenesis through downregulation of vascular endothelial growth factor expression. Zhang et al. demonstrated that combined use of curcumin and homoharringtonine may suppress lymphoma cell growth and blood vessel formation by targeting the VEGF/Akt signaling pathway [60].

Curzerene, a prominent sesquiterpene in *Curcuma* rhizomes, exhibits notable anti-tumor effect in both cellular and animal models. In vitro studies revealed that cuezerene exhibited antiproliferative effects with an IC_50_ value of 47.0 μM over 72 h, causing G2/M phase cell cycle arrest and triggering programmed cell death in SPC-A1 human lung adenocarcinoma cells. Additionally, in vivo experiments showed significantly inhibition of tumor growth in SPC-A1 cell-bearing nude mice treated with curzerene at the dosage of 135 mg/kg/day. The mechanism of anti-tumor activity could be related to the induction of down-regulation of GSTA1 protein and mRNA expression [61]. Demethoxycurcumin, a curcumin derivative, also anti-tumor activity in diverse human cancer cell lines. Kao et al. proved that demethoxycurcumin suppressed cervical cancer progression by modulating PPARγ-mediated pathways, influencing both cellular proliferation and apoptotic processes [62]. Another study suggested that DMC-BH inhibited orthotopic glioma stem cell proliferation via targeting JNK/ERK axis [63]. The above evidence indicated that turmeric and its active components exerted anti-tumor activity mainly through modulation of tumor proliferation, tumor invasion and metastasis, tumor angiogenesis, etc. The mechanisms were involved in targeting PI3K/Akt, JAK/STAT3, Hedgehog/Gli1, PPARγ, JNK/ERK signaling pathways.

#### Neuroprotective activity

Aβ (amyloid-beta) is a protein that accumulates in the brain and forms plaques, which is a hallmark feature of Alzheimer's disease (AD). The accumulation of Aβ plaques is deemed to disrupt cell function and trigger neuroinflammation, leading to cognitive decline and memory loss characteristic of AD. Four curcuminoids derived from turmeric, curcumin, demethoxycurcumin, bisdemethoxycurcumin and (*E*)-1,7**-**bis-(4-hydroxy-phenyl)-1-hepten-3,5-dione—were found to protect PC12 cells from Aβ insult (ED_50_: 0.5–10 μg/mL), compared to the positive control (ED_50_: 37–39 μg/mL). Feng et al. found that the curcumin nanoparticles inhibited Aβ aggregation and promoted Aβ phagocytosis/clearance in microglia. Subsequently, curcumin nanoparticles were endocytosed by microglia and inhibited TLR4/NF-κB pathway for microglia polarization [64]. One study confirmed that the neuroprotective potential of turmeric extract may be mediated by decreasing the levels of malondialdehyde in plasma and brain, and increasing the enzyme activities of SOD, CAT, and GPx in the brain [65]. Then, another study proved that administration of curcumin (50 mg/kg) in a 6-hydroxydopamine-induced parkinson’s disease rat model significantly reduced the aggregation of α-synuclein and improved the parkinsonian disability scores [66]. Besides, intermedin B, isolated and identified as an active compound from turmeric, exhibited neuroprotective effects against HT22 hippocampal cells by reducing inflammation and reactive oxygen species generation [67]. The above studies showed that turmeric demonstrates significant neuroprotective properties by inhibited Aβ aggregation and the generation of ROS, and modulating inflammatory pathways.

#### Hepatoprotective activity

Additionally, turmeric is well-recognized for its hepatoprotective activity. It was demonstrated that the protective effect of curcumin against liver oxidative injury involves restoring gut microbiota balance and lipid metabolism dysregulation caused by Ochratoxin A [68]. Cunningham et al. suggested that curcumin supplementation demonstrated efficacy in decreasing hepatocellular inflammation, hepatic steatosis, NAFLD activity scores, and serum biomarkers associated with liver injury. Totally, in female wistar rat models, curcumin administration improved NASH phenotype, with significant mitigation of hepatocellular inflammation [69]. Furthermore, curcumin supplementation showed the protective effect of hepatic steatosis induced by bisphenol A. The mechanism could potentially regulate gut microbiota homeostasis while reinforcing intestinal barrier integrity, consequently reducing liver inflammatory response triggered by lipopolysaccharide [70]. Formulating curcumin into nanoparticles or liposomes represents a viable strategy to circumvent its inherent bioavailability limitations. Hussain et al. reported that curcumin-incorporated nano-lipid carrier demonstrated hepatoprotective effect in rats with cypermethrin-induced hepatotoxicity [71]. When compared with curcumin, nanoengineered curcumin exhibited enhanced antioxidant capacity and hepatoprotective effect [72].

#### Anti-microbial activity

*Curcuma longa*, known for its anti-microbial activity, showed anti-bacterial activity against *Bacillus subtilis*, *Escherichia coli*, *Pseudomonas aeruginosa*, *Staphylococcus aureus*, and *Vibrio cholerae,* exhibiting MIC values within the range of 125–1000 μg/mL [73]. Also, at 1000 mg/L, the hexane extract of *Curcuma longa* exhibited anti-fungal effect against *Phytophthora infestans*, *Rhizoctonia solani*, and *Erysiphe graminis*, while the ethyl acetate extract of *Curcuma longa* demonstrated fungicidal property against *Botrytis cinerea*, *Puccinia recondita*, *Phytophthora infestans*, and *Rhizoctonia solani* [74]. Additionally, Vetvicka et al. found that five curcumin samples purchased from Sabinsa, Sigma, and Jarrow Formulas showed some anti-*Helicobacter pylori* effects [75]. Lobo de Sá et al. suggested the inhibitory growth activity of curcumin on *Campylobacter jejuni* [76]. Martins et al. reported anti-bacterial activity of curcumin against *Paracoccidioides brasiliensis.* Curcumin significantly reduced the attachment capability of candida species to human buccal epithelial cells [77]. Moreover, *Curcuma longa* also showed anti-viral activity. Curcumin at 40 µM suppressed viral adsorption in an assay, reducing titers by 3.55 log TCID50 ml^−1^, which highlights its anti-adsorption activity against transmissible gastroenteritis virus [78]. Another study showed that *Curcuma longa* extract represses HBV replication through enhancing the level of p53 protein [79]. Then, three curcuminoids compounds, curcumin, demethoxycurcumin and bisdemethoxycurcumin, could serve as potential supplementary agents in preventing and treating diseases caused by influenza viruses. These compounds exhibited the inhibitory activity against novel influenza strains, including H9N2, H1N1, and the oseltamivir-resistant novel H1N1 (H274Y mutant) expressed in 293 T cells [80].

#### Other activities

The synergistic application of a mixture composed of four compounds (curcumin, demethoxycurcumin, bisdemethoxycurcumin and cyclocurcumin) derived from turmeric significantly enhanced anti-nematicidal activity [81]. *ar*-Turmerone and (*E*)-labda-8(17),12-diene-15,16-dial, derived from the volatile oil of turmeric, exhibited potential mosquitocidal and anti-microbial activity, respectively. ar-Turmerone demonstrated a mosquitocidal effect, with an LD_100_ of 50 µg/mL against *Aedes aegyptii* larvae, while (E)-labda-8,12-diene-15,16-dial demonstrated significant anti-fungal efficacy against *Candida parapsilosis* and *Candida kruseii* at a concentration of 25 µg/mL [82]. Drug-metabolizing enzymes, particularly cytochrome P450 enzyme (CYPs), are recognized as significant contributors to adverse drug reactions and therapeutic failures, as they metabolize many currently available therapeutic agents. The sesquiterpene compound (4S,5S)-( +)-germacrone-4,5-epoxide demonstrated significantly enhanced CYP3A4 inhibition compared to curcumin analogs, exhibiting a potent IC_50_ value of 1.0 μM. This inhibitory activity represents a substantial improvement over both curcumin (IC_50_ = 14.9 μM) and demethoxycurcumin (IC_50_ = 7.0 μM), with approximately 15-fold and sevenfold greater potency, respectively. Furthermore, the (4S,5S)-( +)-germacrone-4,5-epoxide compound exhibited the most potent inhibitory activity against CYP1A2, CYP2C9, and CYP3A4 [83]. Ar-turmerone, identified as the main volatile component in turmeric rhizome, demonstrated significant dose-dependent inhibition against both α-glucosidase (IC_50_ = 0.28 μg/mL) and α-amylase (IC_50_ = 24.5 μg/mL) [84]. Additionally, in irinotecan-induced nude mice, curcumin administration significantly alleviated diarrhea symptoms, restored the structural integrity of intestinal mucosa, and upregulated the expression of PRDX4 and P4HB [85]. Moreover, turmeric was reported to possess promising anti-aging activity to maintain healthy aging. It was demonstrated that dietary supplementation with 0.5% turmeric effectively attenuated age-related physiological decline in *Drosophila melanogaster*, primarily through preservation of β-tubulin protein level in cerebral tissue [86].

### Clinical application

Randomized controlled trials (RCTs) are crucial for validating the efficacy of drugs. The current progress of turmeric application in clinical studies demonstrates promising results. In RCTs, turmeric could bring clinical benefits in type 2 diabetes, metabolic syndrome, knee osteoarthritis, hemodialysis, etc. (Table 2). Additionally, In TCM clinical practice, turmeric is typically not used alone but combined with other medicines to form prescriptions.

**Table 2:** Clinical studies of turmeric

Rank	Disease	Sample sizes	Test period	Drug	Result	Ref/NCT no	Refs
1	Knee osteoarthritis (Grade 2 and 3)	60	4 weeks	Curcumin (600 mg/day), gingerols (15 mg/day), piperine (7.5 mg/day)	↓PGE2	IRCT2017070511763 N32	[104]
2	Knee osteoarthritis	144	6 weeks	Turmeric extract (BCM-95®) (1000 mg/day)	↓CRP, TNF-α	CTRI/2017/02/007962	[97]
3	Knee joint pain	68	1 week	B-Turmactive® (Turmeric extracts 500 mg/day + curcuminoid complex 19.5 mg/day)	↓Knee joint pain, CRP	NCT03202901	[105]
4	Hemodialysis	21	12 weeks	Turmeric (3 g/day); turmeric/piperine (3 g turmeric/day + 2 mg piperine/day)	↓MDA, ferritin, GPx	no. 2.594.918	[106]
5	Vitiligo	24	4 months	Turmeric	↓size of lesions; ↑Lesion’s appearance, patient’s satisfaction score	IRCT20180910040994N1	[107]
6	Non-alcoholic fatty liver disease	92	12 weeks	Turmeric supplementation (3 g/day); turmeric and chicory seed supplementation (3 g/day turmeric + infused 9 g/day chicory seed)	↓BMI, WC, (TG/HDL-C)/(LDL-C/HDL-C) ratio; ↑HDL-C	IRCT201406183664N12	[94]
7	Psoriasis	40	9 weeks	Turmeric tonic	↓Erythema, PASI score; ↑patients’ quality of life	IRCT201604183106N30	[108]
8	Premenstrual syndrome	123	3 months	Curcuminoid (500 mg/day)	↓PSST scores, dysmenorrhea pain	IRCT20191112045424N1	[109]
9	Oral submucous fibrosis	35	3 months	Kali Haldi (6 mg/day) + Aloe vera gel (6 mg/day)	↓Burning sensation; ↑cheek flexibility, tongue protrusion	–	[110]
10	Knee osteoarthritis	150	90 days	Turmeric rhizome extract (186.68 or 280.02 mg/day)	↓PGADA, pain, KOOS	ISRCTN12345678	[111]
11	Non-alcoholic fatty liver disease	46	12 weeks	Turmeric powder (3000 mg/d)	↓Glucose, insulin, HOMA-IR, leptin	IRCT201406183664N12	[93]
12	Knee osteoarthritis	101	8 weeks	Curcumin extract (Curcugen®) (1000 mg/d)	↓KOOS knee pain score, numeric knee pain ratings; ↑timed up-and-go test, 6-min walk test, JOA total score	ACTRN12620000976987	[99]
13	Hemodialysis	50	8 weeks	Turmeric capsule (1500 mg/day)	↓MDA; ↑CAT, albumin	–	[112]
14	Self-reported digestive complaints	77	8 weeks	Curcugen™ (500 mg/day)	↓GSRS score, DASS-21 anxiety score	ACTRN12619001236189	[113]
15	Hyperlipidemic type 2 diabetes	75	8 weeks	Powdered rhizome of turmeric (2100 mg/d)	↓BMI, TG, TC	IRCT201204162602	[114]
16	Primary dysmenorrhea	128	–	Turmeric (500 mg/d)	↓Pain	IRCT20141025019669N9	[115]
17	Hemodialysis	100	8 weeks	Turmeric (1500 mg/day,containing 66.3 mg curcumin/day)	↓Hs-CRP, pruritus scores	NCT01037595	[116]
18	Oral cancer (undergone radical surgery)	60	6 weeks	Bio-enhanced turmeric formulation (1 or 1.5 g/day)	↓chemoradiotherapy-induced severe oral mucositis, dysphagia, oral pain, dermatitis	CTRI/2015/12/006413	[101]
19	Oral dysfunctions among head and neck cancer	92	–	–	↓Oral mucositis and associated oral dysfunctions	CTRI/2018/06/014367	[103]
20	Chronic kidney disease (undergoing peritoneal dialysis)	24	12 weeks	Curcumin (1500 mg/day, with 98.42% total curcuminoids)	↓MDA, lipid peroxidation	NCT04413266	[117]
21	Head and neck cancer	80	7 weeks	Turmeric capsule (1200 mg/day)	↓Radiation-induced oral mucositis, intolerable mucositis, body weight	–	[118]
22	Overt type 2 diabetic nephropathy	40	2 months	Turmeric (1500 mg/day, 66.3 mg was curcumin)	↓TGF-β, IL-8, urinary protein excretion	–	[119]
23	Mild to moderate elevated alanine transaminase levels	48	12 weeks	Fermented turmeric powder (3.0 g/day)	↓ALT, AST	NCT01634256	[120]
24	Metabolic syndrome	250	8 weeks	Turmeric (2.4 g/day)	↓LDL-C, CRP	ACTRN12613001053718	[121]
25	Eczema	360	3 months	Indian pennywort, Walnut and Turmeric	Semi quantitative scores of erythema and oedema reduced;itching relieved	–	[122]
26	COVID-19	68	2 weeks	Nanocurcumin (160 mg/day)	↓Coughs, fatigue, myalgia, oxygen demand, oxygen usage, and respiratory rate minimized; ↑SPO_2_	IRCT20211126053183N1	[123]
27	Type 2 diabetes	227	12 months	Curcuminoids (1500 mg/day)	↓Pulse wave velocity, LDL-C, sd LDL-C, CRP, IL-1β, IL-6, TNF-α	TCTR20140303003	[87]
28	Hemodialysis	71	12 weeks	Turmeric (1500 mg/day, containing 66.3 mg curcumin/day)	↓hs-CRP, IL-6, TNF-α; ↑albumin	–	[124]
29	Osteoarthritis	30	3 months	Sinacurcumin® (80 mg/day)	↓Visual Analog Score, CRP, CD4^+^ T cells, CD8^+^ T cells	NCT03715140	[98]
30	Type 2 diabetes	100	12 weeks	Curcumin (1000 mg/day) + piperine (10 mg/day)	↓Leptin, (TNF-α + leptin)/adiponectin ratio; ↑adiponectin	IRCT201505301165N4	[125]
31	Type 2 diabetes	71	120 days	*Curcuma longa* L. (500 mg/d) + piperine (5 mg/d)	↓Glycaemia, glycated haemoglobin, HOMA index, TG	RBR-6r7w8k	[88]
32	Metabolic syndrome with obesity	94	90 days	Calebin A (50 mg/day) + piperine (6 mg/day)	↓Body weight, waist circumference, BMI, LDL-C, TG, leptin, CRP; ↑HDL-C	CTRI/2021/09/036495	[96]
33	Primary knee osteoarthritis	40	6 weeks	Curcuminoid(1500 mg/day) + piperine (15 mg/day)	↑SOD, GSH; ↓MDA	–	[126]
34	Type 2 diabetes	118	12 weeks	Curcuminoids (1000 mg/day) + (piperine 10 mg/day)	↓TC, non-HDL-C, Lp(a); ↑HDL-C	IRCT201505301165N4	[89]
35	Metabolic syndrome	66	12 weeks	curcumin (500 mg/day)	↓Body weight, Pulse wave velocity	IRCT20180619040151N2	[127]
36	Chronic kidney disease	31	3 months	Curcumin (100 mL of orange juice with 12 g of carrot and 2.5 g of turmeric/week)	↓NF-kB mRNA, hsCRP	NCT03475017	[100]
37	Metabolic syndrome	50	12 weeks	Nano-curcumin (80 mg/day)	↓TG, HOMA-β	IRCT20150815023617N3, NCT03534024	[95]
38	Sarcopenia	30	3 months	Cureit™ (500 mg/day)	↑Handgrip strength, weight-lifting capacity	CTRI/2018/05/014176	[128]
39	Laparoscopic gynecologic surgery	60	3 days	Curcuminoid extract (1000 mg/day)	↓Pain severity	TCTR20180215001	[129]
40	Liver cirrhosis	60	3 months	Curcumin (1000 mg/day)	↓Model for end-stage liver disease (MELD) (i), MELD, MELD-Na	IRCT20180802040678N1	[130]
41	Non-alcoholic fatty liver disease	80	2 months	Curcumin (250 mg/day)	↓The grade of hepatic steatosis, AST, hepatic steatosis and enzymes	IRCT2015052322381N1	[131]
42	Polycystic ovarian syndrome	67	3 months	Curcumin (1500 mg/day)	↑PGC1α, Gpx enzyme	IRCT20091114002709N50	[132]
43	Liver cirrhosis	58	12 weeks	Curcumin (1000 mg/day)	↑CLDQ domains, Physical and Mental health (Total) scores, most of SF-36 domains; ↓LDSI 2.0 domains	IRCT20180802040678N1	[133]
44	Type 2 diabetes	229	12 months	Curcumin (1500 mg/day)	↓Fasting blood glucose, HbA1c, HOMA-IR, leptin, BMI; ↑HOMA-β, adiponectin	20140303003	[90]
45	Knee osteoarthritis	140	28 days	Curcuminoid complex (1000 mg/day) + diclofenac (100 mg/day)	↓Pain; ↑quality of life	ISRCTN10074826	134]
46	Type 2 diabetes	114	3 months	*Curcuma longa* L. (400 mg/day)	↓Carotid-femoral pulse wave velocity, left brachial-ankle pulse wave velocity, aortic augmentation pressure, aortic augmentation index, aortic augmentation index at heart rate 75	CTRI/2016/10/007401	[91]
47	Type 2 diabetes	80	8 weeks	Nano-curcumin (80 mg/day)	↓HbA1c, FBS, total score of neuropathy, total reflex score	IRCT20140413017254N5	[92]
48	Nonalcoholic fatty liver diseases	55	8 weeks	Curcuminoids (500 mg/day) + piperine (5 mg/day)	↓Weight, TNF-α, MCP-1, EGF	–	[135]
49	Overweight or prehypertension/mild hypertension	90	12 weeks	*Curcuma longa* L. (900 mg/day)	↓CRP, TNF-α, IL-6, sVCAM-1, glucose, HbA1c, TG; ↑HDL-C	–	[136]
50	Type 2 diabetes	44	10 weeks	Curcumin (1500 mg/day)	↓TG, CRP; ↑adiponectin	NCT02529969	[137]
51	β-thalassemia major	68	12 weeks	Curcumin (1000 mg/day)	↓ NTBI, ALT, AST	IRCT2016053028165N1	[138]
52	Chronic prostatitis/ chronic pelvic pain syndrome type III	48	1 month	Curcumin extract (350 mg) + Calendula extract 80 mg (1 suppository/die/month)	↓NIH-CPSI, IIEF-5, PEDT, peak flow, VAS	–	[139]
53	Cancer	80	8 weeks	Bioavailability-boosted curcuminoids preparation (180 mg/day)	↓TNF-α, TGFβ, IL-6, substance P, hs-CRP, CGRP, MCP-1; ↑Quality of life	–	[102]
54	Osteoarthritis of knee	160	120 days	*Curcuma longa* L. (500 mg/day) + Diclofenac (50 mg/day)	↑IL-1β, ROS, MDA	CTRI/2015/12/006438	[140]
55	Sulfur mustard	89	4 weeks	Curcuminoids (1500 mg/day) + piperine (15 mg/day)	↑GSH, CAT, SGRQ; ↓MDA	–	[141]

#### Treatment of metabolic diseases

Clinical evidences have demonstrated that turmeric supplementation could improve diabetes and its complications. In obese patients with type 2 diabetes individuals receiving curcuminoids supplementation (1500 mg/day) for 12 months, the cardiometabolic risk biomarkers such as small dense low-density lipoprotein cholesterol and low-density lipoprotein cholesterol reduced, along with decreased levels of inflammatory markers including IL-1β, IL-6, hs-CRP, and TNF-α [87]. A recent investigation revealed that turmeric supplementation (500 mg/day with piperine 5 mg/day) within a course of 120 days significantly reduced fasting plasma glucose, glycated hemoglobin, homeostatic model assessment of insulin resistance (HOMA-IR) and triglycerides [88]. Moreover, curcuminoids supplement can reduce the diabetes associated atherogenic risks. In a 12-week intervention study, 59 participants were administered curcuminoids (1000 mg/day with piperine 10 mg/day), while 59 participants were given placebo. The study revealed that curcuminoids can significantly reduce serum concentrations of key atherogenic lipid profiles, such as non-high-density lipoprotein cholesterol (non-HDL-C) and lipoprotein(a) [Lp(a)] [89]. Another study (for 12 months) included 229 individuals indicated that curcuminoids supplementation (1500 mg/day) demonstrated significant improvement in pancreatic β-cell function along with notable reduction in both insulin resistance and body weight compared with the placebo group [90]. Srinivasan et al. showed that 400 mg/day of turmeric intake for 3 months decreased arterial stiffness when compared to that of the placebo group [91]. In addition, it has been reported that nano-curcumin supplementation reduced fasting blood glucose, glycated hemoglobin, total neuropathy score, and total reflex score compared to the placebo group [92].

Furthermore, according to an investigation on the effects of turmeric on serum glucose parameters and leptin levels in patients with nonalcoholic fatty liver disease (NAFLD), 46 individuals were given supplements of 3000 mg/day turmeric powder or placebo for 12 weeks. The findings revealed significant decrease in fasting serum glucose, insulin levels, HOMA-IR scores, and leptin levels [93]. Also, oral turmeric supplementation at a dosage of 3 g/day among patients with non-alcoholic fatty liver disease (NAFLD) led to significant reduction in the serum TG/HDL-C and LDL-C/HDL-C ratio [94]. Besides, in metabolic syndrome patients receiving 80 mg/day nano-curcumin for 12 weeks, the levels of triglyceride and HOMA-β were significantly improved [95]. Another study in metabolic syndrome individuals with obesity, 94 individuals were administered Calebin A, a minor bioactive phytochemical from turmeric*.* The study indicated that Calebin A could significantly reduce circulating leptin and C-reactive protein levels [96]. Overall, studies conducted to date have indicated that turmeric improved the related index of glucolipid metabolism including glycaemia, glycated haemoglobin, HOMA index, insulin resistance, triglycerides, and non-high-density lipoprotein cholesterol, and reduced inflammation.

#### Treatment of inflammatory diseases

Turmeric has proven to be very effective in many types of inflammatory diseases. One study suggested that turmeric extract was as effective as paracetamol in reducing pain and other symptoms associated with knee osteoarthritis. Furthermore, it demonstrated a more favorable safety profile and greater efficacy in lowering inflammatory biomarkers, specifically CRP and TNF-α levels [97]. In another study, thirty patients were randomly assigned to two groups and received either Sinacurcumin^®^ (80 mg daily) or a placebo for a period of three months. The data demonstrated that curcumin significantly decreased visual analogue scale scores, C-reactive protein, and immunological parameters including CD4^+^ and CD8^+^ T cells, Th17 cells and B cells frequency [98]. A follow-up study (for 8 weeks) included 101 individuals at risk of knee osteoarthritis. The study indicated that participants in the Curcugen^®^ (curcumin extract) group experienced significant decreases in their KOOS knee pain scores and numeric knee pain ratings [99]. In chronic kidney disease patients undergoing hemodialysis, a three-month treatment with curcumin supplementation (administered daily as 2.5 g turmeric dissolved in 100 mL orange juice with 12 g carrot) resulted in decreased inflammatory biomarkers, NF-kB mRNA expression and hsCRP protein concentration, suggesting that regular curcumin intake may modulate inflammatory pathways in clinical populations [100].

#### Treatment of cancers

Clinical studies have confirmed the positive clinical efficacy of turmeric in cancers. In a randomized double-blinded placebo-controlled trial (n = 60), researchers evaluated the effects of turmeric formulation capsules in patients with oral cancer. The study revealed that oral administration of curcumin-formulated capsules significantly reduced chemoradiotherapy-induced severe oral mucositis, dysphagia, pain, and dermatitis in oral cancer patients [101]. Another study revealed that curcuminoid supplementation (180 mg/day) within a course of 8 weeks significantly improved the health-related quality of life and suppressed systemic inflammation in patients with solid tumors [102]. In addition, turmeric mouthwash exhibited superior efficacy to benzydamine mouthwash in mitigating both the clinical severity of oral mucositis and related functional impairments among patients undergoing treatment for head and neck carcinoma [103].

## Application of *Curcuma longa* products

*Curcuma longa* has been cherished worldwide because of its medicinal and nutritional value and exhibits broad potential across various industries, such as pharmaceutical industry, food industry, cosmetic industry. Currently, there are 34280 patents related to turmeric worldwide (https://www.lens.org/). The United States has the largest number of patents, accounting for forty-four percent, followed by China. These patents primarily focused on medicine, health food, herbal dietary supplement, cosmetics, and other applications. Based on the *Pharmacopeia of the People’s Republic of China (Edition 2020)* and *Traditional Chinese medicine preparations*, a total of 29 turmeric-containing prescriptions were included, involving Jianghuangxiaocuo liniments, Wujunzhidan tablets, Jiangzhitongluo soft capsules, Wuhuangyangyin particles, Jinfozhitong pills, Yuxuebi capsules, Binghuangfule ointment, Ruyijinhuang powders, Fengtongan capsules, Biwen powders, Jiuweigantai capsules, Xiaotong plasters, Dieda pills, Huazhenghuisheng tablets, Taijishengjiang pills, Zhongmanfenxiao pills, Qingyilidan particles, Shangshijietong plasters, Chansuzhentong plasters, Huangjinboyao wines, Shulereyunji, Guanjiezhentong plasters, Chenxiangshuyu pills, Lidanzhitong tablets, Jingzhiwujiapi wines, Chenxiangshuyu tablets, Yuxuebi particles, Shanhuxuanjing tinctures, and Wudizhitong liniments (Table 3). Among them, Jiangzhitongluo soft capsules is only composed of turmeric extract. The others, such as Jianghuangxiaocuo liniments, Wujunzhidan tablets, Wuhuangyangyin particles, all include many TCM herbs or components, with a wide range of clinical efficacy.

**Table 3:** Prescriptions containing turmeric in TCM clinical applications

Rank	Prescription name	Prescription source	Prescription compositions	Action of prescription
1	Jianghuangxiaocuo liniments	Pharmacopoeia of the People’s Republic of China (Edition 2020)	*Curcuma longa, Paris polyphylla, Polygonum perfoliatum, Chenopodium ambrosioides, Solidago decurrens, Gynostemma pentaphyllum, Zingiber corallinum*	Used for acne caused by damp heat stagnation, and seborrheic dermatitis
2	Wujunzhidan tablets	Pharmacopoeia of the People’s Republic of China (Edition 2020)	*Prunus mume, Rheum officinale, Citrus medica, Citrus aurantium, Origanum vulgare, Gardenia jasminoides, Glycyrrhiza uralensis, Areca catechu, Clematis chinensis, Curcuma longa*	Used for costal pain and biliary distension caused by damp-heat in the liver and gallbladder, presenting with symptoms such as costal rib distending pain, fever, and dark yellow urine; also applicable for patients with cholecystitis, biliary tract infection, or postoperative conditions of the biliary tract who exhibit the above-mentioned symptoms
3	Jiangzhitongluo soft capsules	Pharmacopoeia of the People’s Republic of China (Edition 2020)	*Curcuma longa* extract	Used for hyperlipidemia with Qi stagnation and blood stasis syndrome, characterized by costal and epigastric distending pain, precordial stabbing pain, chest oppression, ecchymosis or petechiae on the tip or margin of the tongue, and string-like or choppy pulse
4	Wuhuangyangyin particles	Pharmacopoeia of the People’s Republic of China (Edition 2020)	*Coptis chinensis, Hedysarum polybotrys, Rehjnannia glutinosa, Curcuma longa, Scutellaria baicalensis*	Used for diabetes mellitus characterized by phlegm-dampness stagnation and Qi-Yin deficiency syndrome, symptoms include polydipsia with frequent drinking, polyphagia with increased appetite, polyuria with frequent urination, heaviness and fatigue in the head and body, nausea and sputum production, fatigue and weakness, shortness of breath and laziness to speak, spontaneous sweating and night sweats, palpitations and insomnia, obesity, dry throat and mouth, irritability and heat intolerance, dark red urine and constipation, etc
5	Jinfozhitong Pills	Pharmacopoeia of the People’s Republic of China (Edition 2020)	*Paeonia lactiflora, Corydalis yanhusuo, Panax notoginseng, Curcuma wenyujin, Citrus medica, Curcuma longa, Glycyrrhiza uralensis*	Used for epigastric pain due to Qi and blood stasis, dysmenorrhea, and pain caused by peptic ulcer and chronic gastritis
6	Yuxuebi capsules	Pharmacopoeia of the People’s Republic of China (Edition 2020)	*Boswellia carterii, Commiphora myrrha, Carthamus tinctorius, Clematis chinensis, Cyathula officinalis, Cyperus rotundus, Curcuma longa, Angelica sinensis, Salvia miltiorrhiza, Ligusticum chuanxiong, Astragalus membranaceus*	Used for arthralgia caused by blood stasis obstructing the collaterals, characterized by severe muscle and joint pain, tenderness on palpation, fixed location, and possible presence of nodules or ecchymosis
7	Binghuangfule ointments	Pharmacopoeia of the People’s Republic of China (Edition 2020)	*Rheum officinale, Curcuma longa, Sulfur, Scutellaria baicalensis, Glycyrrhiza uralensis, Borneolum Syntheticum, Mentholum*	Used for skin itching caused by damp heat accumulation or blood heat and dryness; Pruritic skin diseases such as neurodermatitis, eczema, tinea pedis, and psoriasis are seen in the above syndromes
8	Ruyijinhuang powders	Pharmacopoeia of the People’s Republic of China (Edition 2020)	*Curcuma longa, Rheum officinale, Phellodendron chinense, Atractylodes lancea, Magnolia officinalis, Citrus reticulata, Glycyrrhiza uralensis, Arisaema erubescens, Angelica dahurica, Trichosanthes kirilowii*	Used for erysipelas and furunculosis caused by heat-toxin stagnation in the skin, characterized by erythema (redness), edema (swelling), heat, and tenderness of the skin. It is also applicable for contusions and sprains
9	Fengtongan capsules	Pharmacopoeia of the People’s Republic of China (Edition 2020)	*Stephania tetrandra, Tetrapanax papyrifer, Cinnamomum cassia, Curcuma longa, Gypsum fibrosum, Coix lacryma-jobi, Chaenomeles speciosa, Erythrina variegata, Lonicera japonica, Phellodendron chinense, Talci pulvis, Forsythia suspensa*	Used for arthralgia caused by damp-heat obstructing the collaterals, characterized by erythema, edema (swelling), heat, and tenderness of the joints, as well as myalgia (muscle soreness). It is also applicable for rheumatic arthritis presenting with the above-mentioned symptoms
10	Biwen powders	Pharmacopoeia of the People’s Republic of China (Edition 2020)	*Santalum album, Lysimachia foenum-graecum, Angelica dahurica, Lysimachia capillipes, Curcuma longa, Rosa rugosa, Nardostachys jatamansi, Eugenia caryophyllata, Aucklandia lappa, Artificial moschus, Borneolum syntheticum, Cinnabaris, Mentholum*	Used for dizziness, headache, nasal congestion, nausea, vomiting, motion sickness and seasickness caused by summer heat
11	Jiuweigantai capsules	Pharmacopoeia of the People’s Republic of China (Edition 2020)	*Panax notoginseng, Curcuma wenyujin, Tribulus terrestris, Curcuma longa, Rheum officinale, Scutellaria baicalensis, Scolopendra subspinipes mutilans, Dioscorea opposita, Schisandra chinensis*	Used for costal pain or stabbing pain, depressive symptoms and irritability, anorexia, epigastric fullness and distension after eating, disordered bowel movements, and subcostal masses caused by Qi stagnation and blood stasis combined with liver depression and spleen deficiency
12	Xiaotong plasters	Pharmacopoeia of the People’s Republic of China (Edition 2020)	*Lamiophlomis rotata, Curcuma longa,* etc	Used for acute and chronic sprains, bruises, bone hyperplasia, rheumatism and rheumatoid pain, stiff neck, frozen shoulder, lumbar muscle strain and old injuries
13	Dieda pills	Pharmacopoeia of the People’s Republic of China (Edition 2020)	*Panax notoginseng, Angelica sinensis, Paeonia lactiflora, Prunus persica, Carthamus tinctorius, Daemonorops draco, Siphonostegia chinensis, Drynaria fortunei, Dipsacus asper, Caesalpinia sappan, Paeonia suffruticosa, Boswellia carterii, Commiphora myrrha, Curcuma longa,* etc	Used for traumatic injuries, rupture of muscles and tendons, fractures, hematoma and swelling with pain, acute lumbar sprain
14	Huazhenghuisheng tablets	Pharmacopoeia of the People’s Republic of China (Edition 2020)	*Leonurus japonicus, Carthamus tinctorius, Zanthoxylum bungeanum, Hirudo nipponica, Angelica sinensis, Caesalpinia sappan, Sparganium stoloniferum, Anemone raddeana, Ligusticum chuanxiong, Dalbergia odorifera, Cyperus rotundus, Panax ginseng, Alpinia officinarum, Curcuma longa,* etc	Used for accumulation caused by blood stasis and internal obstruction, dry blood tuberculosis in women, postpartum blood stasis, and abdominal pain and refusal to press
15	Taiji shengjiang pills	Traditional Chinese medicine preparations (Volume 2)	*Bombyx mori, Rheum officinale, Cryptotympana pustulata, Bambusa textilis, Arisaema cum bile, Curcuma longa, Borneolum syntheticum*	Used for pediatric epidemics, fever and convulsions, swollen cheeks, stagnation of milk and food, phlegm and heat constipation
16	Zhongmanfenxiao pills	Traditional Chinese medicine preparations (Volume 3)	*Poria cocos, Polyporus umbellatus, Scutellaria baicalensis, Curcuma longa, Codonopsis pilosula, Atractylodes macrocephala, Pinellia ternata, Citrus reticulata, Anemarrhena asphodeloides, Citrus aurantium*	Used for spleen deficiency and qi stagnation, dampness and heat stagnation, food and lodging water storage, abdominal distension and pain, heat and bitterness, full and noisy, and unfavorable stool
17	Qingyilidan particles	Traditional Chinese medicine preparations (Volume 4)	*Bupleurum chinense, Corydalis yanhusuo, Curcuma longa, Paeonia suffruticosa, Paeonia lactiflora, Ostrea gigas, Lonicera japonica, Rheum officinale*	Used for acute pancreatitis, acute gastritis, and other symptoms
18	Shangshijietong plasters	Traditional Chinese medicine preparations (Volume 5)	*Angelica pubescens, Angelica dahurica, Aconitum carmichaelii, Aconitum kusnezoffii, Cinnamomum tamala, Vaccaria segetalis, Curcuma longa,* etc	Used for myalgia and arthralgia caused by wind-cold-damp pathogens, shoulder and lumbar soreness, joint pain, and traumatic injuries
19	Chansuzhentong plasters	Traditional Chinese medicine preparations (Volume 5)	*Bufo bufo gargarizans, Strychnos nux-vomica, Aconitum carmichaelii, Arisaema erubescens, Realgar, Angelica dahurica, Curcuma longa,* etc	Used for analgesia and dissipation of various swellings, also used for myofascial pain syndrome, osteophytes, osteoarthritis, and other conditions that cause pain
20	Huangjinboyao wines	Traditional Chinese medicine preparations (Volume 6)	*Angelica sinensis, Carthamus tinctorius, Citrus medica, Ligusticum chuanxiong, Illicium difengpi, Myristica fragrans, Cinnamomum cassia, Curcuma longa,* etc	Used for paresthesia of the limbs), myalgia and arthralgia, and epigastric coldness with fullness
21	Shulereyunji	Traditional Chinese medicine preparations (Volume 6)	*Aconitum carmichaelii, Sinapis alba, Angelica pubescens, Ligusticum chuanxiong, Oleum terebinthinae, Oleum eucalypti, Asarum heterotropoides, Artemisia argyi, Atractylodes Lancea, Cinnamomum camphora, Curcuma longa,* etc	Used for myalgia and arthralgia caused by wind-cold-damp stagnation, lumbar muscle pain, lumbar muscle strain, periarthritis of the shoulder, and rheumatic arthritis
22	Guanjiezhentong plasters	Traditional Chinese medicine preparations (Volume 7)	*Capsicum annuum, Cinnamomum cassia, Aconitum carmichaelii, Aconitum kusnezoffii, Asarum heterotropoides, Curcuma longa,* etc	Used for myalgia, arthralgia, and soft tissue injuries such as sprains
23	Chenxiangshuyu pills	Traditional Chinese medicine preparations (Volume 10)	*Aquilaria sinensis, Aucklandia lappa, Magnolia officinalis, Citrus aurantium, Corydalis yanhusuo, Amomum kravanh, Citrus reticulata, Amomum villosum, Cyperus rotundus, Citrus reticulata, Bupleurum chinense, Curcuma longa*, etc	Used for epigastric fullness and bloating, epigastric pain, vomiting of acid, indigestion, anorexia, and dysphoria
24	Lidanzhitong tablets	Traditional Chinese medicine preparations (Volume 11)	*Isatis indigotica, Taraxacum mongolicum, Artemisia scoparia, Curcuma longa, Melia toosendan, Bupleurum chinense, Paeonia lactiflora, Corydalis yanhusuo, Citrus aurantium, Atractylodes Lancea, Agrimonia pilosa, Glycyrrhiza uralensis*	Used for costal pain and jaundice caused by damp-heat in the liver and gallbladder, such as in acute and chronic hepatitis and cholecystitis
25	Jingzhiwujiapi wines	Traditional Chinese medicine preparations (Volume 13)	*Carthamus tinctorius, Curcuma longa, Citrus reticulata, Polygonatum odoratum, Acanthopanax gracilistylus, Chaenomeles speciosa, Achyranthes bidentata, Codonopsis pilosula, Santalum album, Rehjnannia glutinosa*, etc	Used for hepatic and renal deficiency, muscular and skeletal atrophy, rheumatism and arthralgia muscle and joint contracture, paresthesia of the limbs, lumbar and leg soreness, and epigastric fullness and discomfort
26	Chenxiangshuyu tablets	Traditional Chinese medicine preparations (Volume 15)	*Aquilaria sinensis, Aucklandia lappa, Magnolia officinalis, Amomum villosum, Amomum kravanh, Citrus aurantium, Bupleurum chinense, Corydalis yanhusuo, Cyperus rotundus, Curcuma longa,* etc	Used for epigastric fullness and bloating, epigastric pain, vomiting of acid, indigestion, anorexia, and dysphoria
27	Yuxuebi particles	Traditional Chinese medicine preparations (Volume 16)	*Clematis chinensis, Cyathula officinalis, Boswellia carterii, Commiphora myrrha, Carthamus tinctorius, Salvia miltiorrhiza, Ligusticum chuanxiong, Angelica sinensis, Curcuma longa, Astragalus membranaceus, Cyperus rotundus*	Used for arthralgia due to blood stasis obstructing the collaterals
28	Shanhuxuanjing tinctures	Traditional Chinese medicine preparations (Volume 18)	*Zingiber corallinum, Curcuma longa, Borneolum syntheticum, Salicylic acid, Acetic acid,* etc	Used for tinea pedis, tinea manus, and onychomycosis
29	Wudizhitong liniments	Traditional Chinese medicine preparations (Volume 19)	*Aconitum carmichaelii, Aconitum kusnezoffii, Arisaema erubescens, Carthamus tinctorius, Curcuma phaeocaulis, Borneolum syntheticum, Boswellia carterii, Commiphora myrrha, Curcuma longa, Eugenia caryophyllata, Asarum heterotropoides, Paeonia ladiflora*	Used for acute and chronic sprains and contusions, and chilblains

In the food industry, the health-promoting properties of turmeric rhizomes have garnered considerable attention. According to the State Administration for Market Regulation, there are totally 63 health foods containing turmeric, including 41 capsules, 18 tablets, 1 particle, 1 powder, 1 drink, and 1 tea, such as haishenjianghuang soft capsule, jianghuanghuangqi tablet, jianghuangbaishaorenshen particle, jianghuangyuganzihezi powder and others. Furthermore, turmeric is also used as food additives. According to the “GB 2760–2014 National Food Safety Standard for Use of Food Additives” in China, the maximum allowable amount of curcumin for frozen drinks is 0.15 g/kg, for cocoa products, chocolate & chocolate products (including chocolate & chocolate products with cocoa butter alternatives), and candies is 0.01 g/kg, for batter, coating flour, and fried powder is 0.3 g/kg, for instant noodles products and seasoning syrup is 0.5 g/kg, for compound seasoning is 0.1 g/kg, carbonated drinks is 0.01 g/kg (GB 2760–2014 National Food Safety Standard for Use of Food Additives). Besides, it is worth noting that in most western countries, Turmeric has been extensively regarded as dietary supplement for diverse conditions, including arthritis, respiratory infections, digestive disorders, depression, liver disease, allergies, and many others, with many forms, such as tablet, capsule, particle, soft gel, powder, bar, liquid, etc. In the US mainstream multi-outlet channel, total sales of turmeric increased steadily from 2013 to 2022. In the US natural channel, turmeric was the top-selling primary ingredient from 2013 until 2018, when it dropped to the second position due to a surge of interest in cannabidiol. In 2022, turmeric regained its top rank in the natural channel.

In the cosmetics industry, turmeric has gained considerable popularity due to its potent anti-inflammatory activity, anti-oxidant activity, and anti-bacterial activity. According to records, turmeric is considered one of the earliest cosmetic because it traditionally has been smeared on the skin by Indian women [142]. One study suggested that a nanosphere loaded with curcumin enhanced the mobilization of umbilical cord blood-derived mesenchymal stem cells (UCB-MSCs), thereby promoting cutaneous wound repair [143]. Additionally, in vitro testing on ‘pumpless skin-on-a-chip’ with turmeric leaf extract, the enhancement of the epidermal barrier function demonstrated significant anti-aging efficacy [144]. The “Catalogue of Used Cosmetic Ingredients (2021 Edition)” released by the National Medical Products Administration includes over 8,965 used cosmetic ingredients, with 10 cosmetic ingredients related to turmeric, including *curcuma longa* root, *curcuma longa* root powder, *curcuma longa* rhizome extract, *curcuma longa* root water, *curcuma longa* root extract, *curcuma longa* root oil, *curcuma longa* extract, *curcuma longa* leaf extract, curcumin, and tetrahydrocurcumin (The “Catalogue of Used Cosmetic Ingredients (2021 Edition)”). Today, Cosmetics brands containing turmeric are spread all over the world, as CN formulator, boben, home’ jubi lant and voolga in China, origins, sunday riley, first aid beauty and Kiehl’s in the United States of America, forest essentials and himalaya in India.

## Future perspectives

**Fig. 4:**
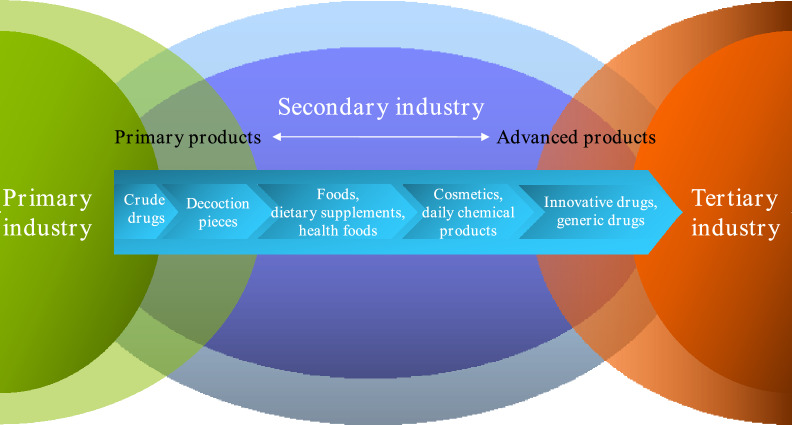
Related industries and products of turmeric

Considering the applications of turmeric in various fields around the world, the establishment of an international standard for turmeric rhizome is necessary to guarantee the clinical effectiveness, safety and controllability in global commerce and trade. Our team and professor Wang Mei from Leiden university jointly initiate the proposal of the ISO international standard “ISO 9299: 2024 Traditional Chinese medicine-*Curcuma longa* rhizome”, which applies to the cultivation and commercialization of cultivated turmeric rhizome that is commercially traded and utilized globally as natural medicine, including Chinese materia medica (whole medicinal materials) and processed decoction pieces derived from its rhizome. In addition, based on the turmeric-related research and systematic summary of the fourth national survey of Chinese materia medica resources, we proposed a concept and scientific connotation of generalized science of Chinese material madica, and established a comprehensive framework, the cultivation system of large varieties of Chinese medicinal materials and the application system of the large health industries [145]. Taking turmeric as the subject, turmeric is involved in the three major industries and related products, from primary products to advanced products, including crude drugs (chinese medicinal materials), decoction pieces, foods, dietary supplements, health foods, cosmetics, daily chemical products, innovative drugs, generic drugs, etc. (Fig. 4). Additionally, application of new extraction techniques and delivery systems have brought regulatory challenges. As the products must satisfy rigorous quality control protocols and safety regulations, which often vary significantly across regions, so trans-regional cooperation is needed.

## Data Availability

Not applicable.

## Abbreviations

Akt: Protein kinase B
ARE: Anti-oxidative response element
CAT: Catalase
CRP: C-reactive protein
DSS: Dextran sulfate sodium
ERK: Extracellular signal-regulated kinase
GPx: Glutathione peroxidase
GR: Glutathione reductase
GSH: Glutathione
GSTA1: Glutathione S-Transferase Alpha 1
H2O2: Hydrogen peroxide
HBV: Hepatitis B virus
HDL-C: High density lipoprotein cholesterol
hs-CRP: High-sensitivity-C-reactive protein
IKK: Inhibitor of kappa B kinase
IL-1β: Interleukin-1β
IL-6: Interleukin-6
IL-8: Interleukin-8
IR: Insulin resistance
IκBα: Nuclear factor kappa-B inhibitor α
JAK: Janus kinase
JNK: c-Jun N-terminal kinase
Keap1: Kelch-like ECH-associated protein 1
LDL-C: Low-density lipoprotein cholesterol
LPS: Lipopolysaccharides
MAPKs: Mitogen-activated protein kinases
MMP-1: Matrix metalloproteinases-1
MMP-3: Matrix metalloproteinases-3
MMP-9: Matrix metalloproteinases-9
mTOR: Mammalian target of rapamycin
NAFLD: Non-alcohol fatty liver disease
NASH: Nonalcoholic steatohepatitis
NF-κB: Nuclear factor kappa-B
NLRP3: NOD-like receptor protein 3
NO: Nitric oxide
Nrf2: Nuclear factor erythroid 2-related factor 2
PI3K: Phosphatidylinositol 3-kinase
PPARγ: Peroxisome proliferator-activated receptor γ
ROS: Reactive oxygen species
SIRT3: Sirtuin 3
SOD: Superoxide dismutase
STAT: Signal transducers and activators of transcription
TG: Triglyceride
TLR4: Toll-like receptor 4
TNF-α: Tumor necrosis factor-α
TREM2: Triggering receptor expressed on myeloid cells 2
